# Prediction model for cognitive frailty in older adults: A systematic review and critical appraisal

**DOI:** 10.3389/fnagi.2023.1119194

**Published:** 2023-04-12

**Authors:** Jundan Huang, Xianmei Zeng, Mingyue Hu, Hongting Ning, Shuang Wu, Ruotong Peng, Hui Feng

**Affiliations:** ^1^Xiangya School of Nursing, Central South University, Changsha, China; ^2^Oceanwide Health Management Institute, Central South University, Changsha, China; ^3^National Clinical Research Center for Geriatric Disorders, Xiangya Hospital, Central South University, Changsha, China

**Keywords:** cognitive frailty, prediction models, older adults, systematic review, critical appraisal

## Abstract

**Background:**

Several prediction models for cognitive frailty (CF) in older adults have been developed. However, the existing models have varied in predictors and performances, and the methodological quality still needs to be determined.

**Objectives:**

We aimed to summarize and critically appraise the reported multivariable prediction models in older adults with CF.

**Methods:**

PubMed, Embase, Cochrane Library, Web of Science, Scopus, PsycINFO, CINAHL, China National Knowledge Infrastructure, and Wanfang Databases were searched from the inception to March 1, 2022. Included models were descriptively summarized and critically appraised by the Prediction Model Risk of Bias Assessment Tool (PROBAST).

**Results:**

A total of 1,535 articles were screened, of which seven were included in the review, describing the development of eight models. Most models were developed in China (*n* = 4, 50.0%). The most common predictors were age (*n* = 8, 100%) and depression (*n* = 4, 50.0%). Seven models reported discrimination by the C-index or area under the receiver operating curve (AUC) ranging from 0.71 to 0.97, and four models reported the calibration using the Hosmer–Lemeshow test and calibration plot. All models were rated as high risk of bias. Two models were validated externally.

**Conclusion:**

There are a few prediction models for CF. As a result of methodological shortcomings, incomplete presentation, and lack of external validation, the models’ usefulness still needs to be determined. In the future, models with better prediction performance and methodological quality should be developed and validated externally.

**Systematic review registration:**

www.crd.york.ac.uk/prospero, identifier CRD42022323591.

## Introduction

According to the World Health Organization, by 2030, older adults will increase to 1.4 billion (World Health Organization [WHO], 2021). Aging contributes to many chronic conditions, such as frailty and cognitive impairment (Robertson et al., 2013). In 2013, the International Consensus Group from the International Academy of Nutrition and Aging (IANA) and the International Association of Gerontology and Geriatrics (IAGG) reached a consensus on the concept of cognitive frailty (CF). CF is the presence of physical frailty and cognitive impairment (clinical dementia score of 0.5) with the absence of dementia (Kelaiditi et al., 2013). Due to the difference in assessment tools and criteria, the prevalence of CF ranged from 1.2 to 22.0% in community-dwelling older adults (Arai et al., 2018). One study focused on community-dwelling older adults indicated that the pooled prevalence of CF was 9.0% (Qiu et al., 2022). CF may contribute to a range of adverse outcomes (Feng et al., 2017; Zhang et al., 2021), for instance, dementia, disability, low quality of life, depression, and mortality.

CF includes two subtypes: reversible and potentially reversible CF, depending on the degree of cognitive impairment. The cognitive impairment of potentially reversible CF is mild cognitive impairment, while reversible CF is usually subjective cognitive decline. Studies have shown that if an early intervention was implemented, the CF could be reversed (Ruan et al., 2015; Merchant et al., 2021). However, there are no unified diagnostic tools for CF. Most previous studies have been conducted by trained clinicians and used comprehensive but time-consuming tools to assess cognitive function (Sugimoto et al., 2018). It is recommended to develop prediction models to identify high-risk individuals (Chen et al., 2022).

There are some prediction models on CF. Yang and Zhang (2021) developed a prediction model for CF in community-dwelling older adults with chronic diseases. Predictors included age, living alone, physical exercise, nutrition, and depression. The C-index of the model was as high as 0.97. However, this model did not validate externally. Tseng et al. (2019) also developed a prediction model for CF among community-dwelling older adults. Predictors included age, gender, waist circumference, calf circumference, memory deficits, and diabetes mellitus. However, the definition of CF varied in the development and validation datasets, which could cause the risk of bias (ROB). The existing models varied in predictors and prediction performances, and the methodological quality of these models still needs to be determined.

Therefore, this systematic review aims to summarize the prediction models for CF, assess their methodological quality, and provide some insight for future research.

## Methods

This systematic review was designed according to the Preferred Reporting of Items in Systematic Reviews and Meta-Analyzes (PRISMA) guidance (Page et al., 2021). A protocol for this study had been registered in the PROSPERO (CRD42022323591).

### Eligibility criteria

Inclusion criteria: (1) Studies’ participants were older adults (60 years older and over); (2) studies reported developing or validating at least one multivariable model for predicting reversible or potentially reversible CF or both; (3) studies had the outcome of interest as CF; (4) studies published in English or Chinese; and (5) Studies were conducted in institutionalized, hospitalized, or community-dwelling settings.

Exclusion criteria: (1) studies were reviews or case reports; (2) studies provided insufficient data for pooling despite several attempts to contact authors; and (3) studies were univariate prediction models.

### Literature search

Through preliminary literature search and expert consultation, we systematically searched PubMed, Embase, Cochrane Library, Web of Science, Scopus, PsycINFO, CINAHL, China National Knowledge Infrastructure (CNKI), and Wanfang Databases from the inception to March 1, 2022. The following MeSH terms and free words were used: “aging,” “older adults,” “cognitive frailty,” “cognitive impairments,” “frailty,” “clinical decision rules,” “prediction model,” “risk model,” and so on. The detailed search strategy and results are provided in the Supplementary appendix. Additionally, we manually searched citations for potential studies.

### Literature selection

Two reviewers (JH and XZ) independently assessed the titles and abstracts of studies for inclusion. Both authors compared selected studies for inclusion. Discrepancies were resolved through discussion. If there are still discrepancies, a third senior researcher (MH) will join in to discuss and make a consensus. After this initial screening, the full-text articles were retrieved for all records. Full-text articles were also screened independently by two reviewers (JH and XZ).

### Data extraction

One reviewer (JH) independently extracted the data, while another (XZ) checked the extracted data. The two reviewers resolved discrepancies through discussion. If there are still discrepancies, a third senior researcher (MH) will join in to discuss and make a consensus.

Data were extracted using a form based on the Checklist for Critical Appraisal and Data Extraction for Systematic Reviews of Prediction Modelling Studies (CHARMS) (Moons et al., 2014). Extracted information on each study included: (1) basic information (e.g., first author, year of publication); (2) outcome measurement methods; (3) source of data; (4) participants (age, gender, country); (5) outcomes to be predicted; (6) predictors; (7) event per variable (EPV); (8) missing data and handling methods; (9) modeling methods; (10) model performances (discrimination, calibration, and classification); (11) model evaluation, and (12) model presentation. An EPV was calculated as overfitting for model development. Studies for model development with EPVs less than 10, while for model validation with participants less than 100, are likely to be overfitting (Wolff et al., 2019).

Model performances were assessed by discrimination, calibration, and classification. Discrimination, measured by the C-index and area under the receiver operating characteristic curve (AUC), is the extent to distinguish those at higher or lower risk of having an event. Calibration refers to the degree of agreement between predicted and observed risks, measured by calibration plot, calibration slope, and Hosmer–Lemeshow test (Alba et al., 2017). The AUC or C-index of 0.7–0.8 is acceptable, 0.8–0.9 is excellent, and more than 0.9 is outstanding (Mandrekar, 2010).

Model validation includes internal and external validation. Internal validation aims to test the reproducibility of the model based on the development cohort data. While external validation is to evaluate the model prediction performance in the new data, focusing on model transportability and generalizability (Royston and Altman, 2013).

When a study included multiple models for the same population and outcome, the model with the best performance was extracted for data extraction. When a study developed multiple models, separate data extraction was performed for each model.

### Risk of bias and application assessment

Two reviews (JH and XZ) independently assessed the ROB and application of included studies using the Prediction Model Risk of Bias Assessment Tool (PROBAST) (Moons et al., 2019; Wolff et al., 2019). Discrepancies were resolved through discussion. If there are still discrepancies, a third senior researcher (MH) will join in to discuss and make a consensus. PROBAST consists of 4 domains and 20 signaling questions for ROB assessment. Each signaling question is answered with YES (Y), PROBABLY YES (PY), NO INFORMATION (NI), PROBABLY NO (PN), or NO (N). In a domain, this area can be rated as a low risk only when the answers to all questions are Y or PY. A domain containing at least one question rated as N or PN will be considered high risk. Otherwise, it will be deemed to be at unclear risk. The overall ROB is graded as low risk when all domains are regarded as low risk, and the overall ROB is considered high risk when at least one of the domains is deemed high risk. Otherwise, it will be deemed to be at unclear risk. The application assessment is similar to the ROB assessment but includes only three domains.

### Statistical analysis

Due to the differences in the definition criteria of CF, types of predictors, modeling methods, and characteristics of participants, we just calculated and reported descriptive statistics to summarize the characteristics of the models without any quantitative synthesis.

## Results

### Study selection

**FIGURE 1:**
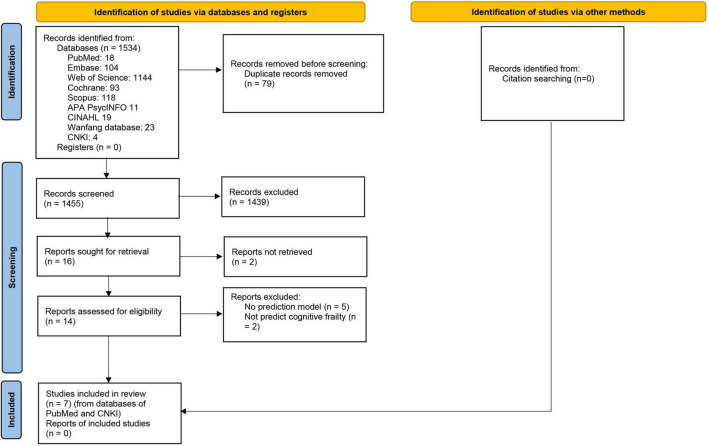
PRISMA 2020 flow diagram for literature screening and selection.

This review searched 1535 records, and only seven studies (Tseng et al., 2019; Navarro-Pardo et al., 2020; Rivan et al., 2020; Sargent et al., 2020; Wen et al., 2021; Yang and Zhang, 2021; Chen et al., 2022) were eligible (Figure 1), including eight prediction models. Among these models, seven were diagnostic, and only one was prognostic.

### Study designs and study populations

Included study characteristics are summarized in Tables 1, 2. Most of the models were developed using data from cross-sectional studies (*n* = 7, 87.5%), and most of the participants were from China (*n* = 4, 50.0%) (Tseng et al., 2019; Wen et al., 2021; Yang and Zhang, 2021; Chen et al., 2022), while the others were from Spain (Navarro-Pardo et al., 2020) (*n* = 1, 12.5%), Malaysia (Rivan et al., 2020) (*n* = 1, 12.5%) and Italy (Sargent et al., 2020) (*n* = 1, 12.5%).

**TABLE 1:** Characteristics of included studies.

References	Type of model (D/V)	Study design	Participants				Candidate predictors	EPV	Outcome	Outcome measurement	Outcome incidence or prevalence	Methods to handle missing value	Model development; model presentation
			**No. of events/ Participants**	**Age**	**Male/ Female**	**Country**							
Chen et al., 2022	D, V	Cross-sectional study	138/368	60∼91	237/289	China	Age, gender, education, marital status, physical exercise, time for consistent exercise, intellectual activity, social activity, self-assessment of sleep quality, nocturnal sleep duration (h), chronic pain; history of falls, daytime mental state, the number of chronic diseases, number of medications taken, self-rated health, fasting blood-glucose, traditional Chinese medicine body composition, nutritional status, IADL, and family functions.	6.57	Both reversible and potentially reversible CF	Frailty phenotype, MoCA, and CDR	Prevalence: 37.5%	No missing data	Logistic regression and Nomogram
Navarro-Pardo et al., 2020	D	Cross-sectional study	62/285	60∼89	132/153	Spain	Age, gender, formal education, profession, social support status, and psychological wellbeing.	8.86	Potentially reversible CF	MoCA and modified version of frailty phenotype	Prevalence: 24.03%	No missing data	Logistic regression and NR
Rivan et al., 2020	D	Prospective cohort study	100/282	67.00 (4.98)	126/156	Malaysia	Socio-demographic and health information, nutritional status, body composition, blood pressure, cognitive function, physical function, dietary intake, psychosocial factors, and biochemical indices.	4.17	Potentially reversible CF	Fried criteria, Petersen and Lee criteria	Incidence: 35.5%	No handling missing data	Logistic regression and NR
Sargent et al., 2020	D	Cross-sectional study	257/1155	Control group: 73 (0.22) and CF: 82 (0.41)	500/655	Italy	132 SNPs and 155 protein biomarker variables, age, sex, level of education, anticholinergic burden, depressive symptoms, baseline diagnosis of dementia, and vascular dementia.	0.87	Potentially reversible CF	Fried criteria and MMSE	Prevalence: 22.3%	No handling missing data	Machine learning (xgboost) and NR
Sargent et al., 2020	D	Cross-sectional study	412/1145	Control group: 61 (0.50) and CF: 76 (0.67)	522/522	Italy	132 SNPs and 155 protein biomarker variables, age, sex, level of education, anticholinergic burden, depressive symptoms, baseline diagnosis of dementia, and vascular dementia.	1.40	Potentially reversible CF	Fried criteria, TMT-A, and TMT-B	Prevalence: 36.0%	No handling missing data	Machine learning (xgboost) and NR
Tseng et al., 2019	D, V	Cross-sectional study	155/724	73.1 (5.4)	385/339	China	Age, sex, body mass index, waist circumference, calf circumference, visual acuity, education, tobacco smoker, take alcohol, diabetes mellitus, hypertension, dyslipidemia, and coronary artery disease.	14.09	Potentially reversible CF	6-metre walk speed, handgrip strength, and multiple neuropsychological assessments	Prevalence: 21.4%	No missing data	Binary logistic regression and Risk score
Wen et al., 2021	D	Cross-sectional study	101/848	60∼88	541/307	China	Subjective symptoms, exhaustion, weight loss, memory complaints, depression, functional assessment, gait speed (m/s),and gait speed (m/s)	11.22	Potentially reversible CF	Fried phenotype, MoCA, and CDR	Prevalence: 11.9%	No handling missing data	Logistic regression and Nomogram
Yang and Zhang, 2021	D	Cross-sectional study	226/674	60∼89	287/387	China	Age, education, the number of chronic diseases, living alone, sleep quality, physical exercise, intellectual activity, nutrition, and depression.	25.11	Potentially reversible CF	Fried phenotype, MoCA, and CDR	Prevalence: 33.5%	No handling missing data	Logistic regression and Nomogram

D, development; V, validation; EPV, event per variable; CF, cognitive frailty; IADL, instrumental activities of daily living scale; MoCA, montreal cognitive assessment; CDR, clinical dementia rating; TUG, timed-Up-and-Go; GDS, geriatric depression scale; NR, not reported; SNPs, single nucleotide polymorphisms; MMSE, mini-mental state examination; TMT, trail making tests.

**TABLE 2:** Validation methods and predictive performance of included studies.

References	Validation method		Predictive performance				
	**Internal validation**	**External validation**	**Discrimination: C index or AUC (95% CI)**	**Calibration**	**Classification**		
					**Sensitivity**	**Specificity**	**Accuracy**
Chen et al., 2022	Bootstrap	Temporal validation	Internal 0.910 (0.863∼0.936) and external 0.850 (0.785∼0.915)	Calibration plot (showed satisfactory fitness) and Brier score 0.117	79.7%	89.1%	85.6%
Navarro-Pardo et al., 2020	NR	NR	NR	*P* = 0.36	NR	NR	76.6%
Rivan et al., 2020	NR	NR	0.826 (NR)	NR	81.1%	76.1%	NR
Sargent et al., 2020	Random split and bootstrap	NR	0.88 (0.83∼0.90)	NR	NR	NR	NR
Sargent et al., 2020	Random split and bootstrap	NR	0.86 (0.80∼0.90)	NR	NR	NR	NR
Tseng et al., 2019	Bootstrap	Geographic validation	Internal 0.71 (NR) and external 0.69 (NR)	*P* = 0.48	70%	60%	NR
Wen et al., 2021	Bootstrap	NR	0.835 (0.771∼0.899)	*P* = 0.103	NR	NR	NR
Yang and Zhang, 2021	Bootstrap	NR	0.970 (0.995∼0.986)	*P* = 0.985	NR	NR	NR

AUC, area under the receiver operating curve; CI, confidence interval; NR, not reported; *P*, the *p*-value of the Hosmer–Lemeshow test.

All models were developed for older adults aged 60–91 years. Furthermore, there was no specific gender.

### Outcome prevalence or incidence

Only one model measured outcomes of potentially reversible and reversible CF (Chen et al., 2022), and the remainings measured potentially reversible CF. The prevalence of potentially reversible and reversible CF was 37.5%, while the prevalence of potentially reversible CF ranged from 11.91 to 36.0%. One study suggested that the incidence of CF was 35.5% (Rivan et al., 2020).

### Predictors

All prediction models reported their predictors. The number of predictors ranged from four to seven. Only two (Sargent et al., 2020) had more than 50 predictors because of the inclusion of single nucleotide polymorphisms (SNPs), protein biomarkers, and other covariates. Most of the models included similar predictors, for example, age (*n* = 8, 100%), depression (*n* = 4, 50.0%), physical exercise (*n* = 3, 37.5%), education (*n* = 4, 50.0%), and chronic disease (*n* = 3, 37.5%).

### Sample size

All models reported the number of participants included in the final prediction model. The number of participants for model development ranged from 282 to 1155, and EPV ranged from 0.87 to 25.11. Among the eight prediction models, one prediction model (Yang and Zhang, 2021) (12.5%) had an EPV of more than 20, two prediction models (Tseng et al., 2019; Wen et al., 2021) (20.0%) had an EPV between 10 and 20, and the remaining five prediction models (Navarro-Pardo et al., 2020; Rivan et al., 2020; Sargent et al., 2020; Chen et al., 2022) (62.5%) had an EPV less than 10.

### Modeling method

Logistic regression (*n* = 6, 75.0%) was the most common modeling method (Tseng et al., 2019; Navarro-Pardo et al., 2020; Rivan et al., 2020; Wen et al., 2021; Yang and Zhang, 2021; Chen et al., 2022) among the included models, while the other two prediction models (Sargent et al., 2020) were developed by machine learning (*n* = 2, 25.0%).

### Model performance

Seven prediction models reported AUC or C-index, ranging from 0.71 to 0.970. Specifically, two models showed outstanding (Yang and Zhang, 2021; Chen et al., 2022), three showed excellent, and one showed acceptable discrimination (Tseng et al., 2019; Rivan et al., 2020; Sargent et al., 2020). Five prediction models reported calibration using the Hosmer–Lemeshow test (Tseng et al., 2019; Navarro-Pardo et al., 2020; Wen et al., 2021; Yang and Zhang, 2021) (*n* = 4, 50.0%) and the calibration plot and Brier score (Chen et al., 2022) (*n* = 1, 12.5%), while the remaining three prediction models did not report calibration.

The classification includes sensitivity, specificity, and accuracy. Three models reported the sensitivity ranging from 70.0 to 81.1%, and the specificity ranging from 60 to 89.1%. One model only reported the accuracy of 76.6% (Navarro-Pardo et al., 2020).

### Model evaluation

Four prediction models were internally validated using bootstrapping (Tseng et al., 2019; Wen et al., 2021; Yang and Zhang, 2021; Chen et al., 2022) (50.0%), and the other two (Sargent et al., 2020) were validated by the random split method and bootstrapping. Only two (Navarro-Pardo et al., 2020; Rivan et al., 2020) prediction models did not report whether internal validation was performed (25.0%). Notably, only two models were externally validated (25.0%).

### Model presentation

Only four models presented the final models using nomogram (Wen et al., 2021; Yang and Zhang, 2021; Chen et al., 2022) (*n* = 3, 37.5%) and risk score (Tseng et al., 2019) (*n* = 1, 12.5%), while the remaining four (Navarro-Pardo et al., 2020; Rivan et al., 2020; Sargent et al., 2020) (50.0%) did not report the presentation.

### Risk of bias and application assessment

All models were evaluated as high ROB. In the outcome domain, one model (Rivan et al., 2020) was judged as high ROB because predictors were not excluded from the outcome definition. In the analysis domain, all models were at high ROB, mainly due to the small sample size, failure to handle missing data, selecting predictors by univariable analysis, and inappropriate ways to categorize continuous predictors.

In terms of application, two models (Sargent et al., 2020) had a high concern in the predictor domain. They were developed using SNPs and protein biomarkers to understand the underlying biological mechanisms for the relationship between physical frailty and cognitive impairment, so they were probably not suitable for screening CF in older adults.

Details of the ROB and applicability of all studies were presented in Table 3 and Supplementary Table 1.

**TABLE 3:** ROB and application assessment of cognitive frailty prediction models.

References	ROB				Application			Overall	
	**Participants**	**Predictors**	**Outcomes**	**Analysis**	**Participants**	**Predictors**	**Outcomes**	**ROB**	**Application**
Chen et al., 2022	**+**	**+**	**+**	**–**	**+**	**+**	**+**	**–**	**+**
Navarro-Pardo et al., 2020	**+**	**+**	**+**	**–**	**+**	**+**	**+**	**–**	**+**
Yang and Zhang, 2021	**+**	**+**	**+**	**–**	**+**	**+**	**+**	**–**	**+**
Wen et al., 2021	**+**	**+**	**+**	**–**	**+**	**+**	**+**	**–**	**+**
Sargent et al., 2020	**+**	**+**	**+**	**–**	**+**	**–**	**+**	**–**	**–**
Sargent et al., 2020	**+**	**+**	**+**	**–**	**+**	**–**	**+**	**–**	**–**
Rivan et al., 2020	**+**	**+**	**–**	**–**	**+**	**+**	**+**	**–**	**+**
Tseng et al., 2019	**+**	**+**	**+**	**–**	**+**	**+**	**+**	**–**	**+**

ROB, risk of bias; “+” low risk; “-” high risk; “?” unclear.

### Model comparison

We compared all models to assess their performances and predictors. More details were shown in Table 4.

**TABLE 4:** Predictors, advantages, and disadvantages of included studies.

References	Predictors	Advantages	Disadvantages
Chen et al., 2022	IADL, self-evaluation of health, self-evaluation of daytime mental state, Number of chronic diseases, age, nutritional condition, and physical exercise	● Relatively easy to measure ● Contains few predictors ● Performance is excellent ● Higher applicability and accessibility ● Externally validated.	● Subjective report information may be inconsistent with the real situation ● Sample size of the model is limited
Navarro-Pardo et al., 2020	Age, formal education, profession, and psychological wellbeing	● Relatively easy to measure ● Contains few predictors	● Limited testing of model performance ● No internal and external validation
Rivan et al., 2020	Age, digit symbol, TUG test, GDS, vitamin D, and physical frailty	● Contains few predictors ● Performance is excellent	● Need to measure vitamin D ● No external validation
Sargent et al., 2020	SNPs, protein biomarkers, gender, age, education, baseline diagnosis of dementia, vascular dementia, depression, and Parkinson’s disease	● Contains few predictors ● Performance is excellent	● Need to measure SNPs and protein biomarkers ● No external validation
Sargent et al., 2020	SNPs, protein biomarkers, gender, age, education, baseline diagnosis of dementia, vascular dementia, depression, and Parkinson’s disease	● Contains few predictors ● Performance is excellent	● Need to measure SNPs and protein biomarkers ● No external validation
Tseng et al., 2019	Age, gender, waist circumference, calf circumference, memory deficits, and diabetes mellitus	● Relatively easy to measure ● Contains few predictors ● Easy to implement in community settings ● Externally validated.	● Performance is acceptable
Wen et al., 2021	Age, sleep events per night, history of diabetes, heart failure, history of hypertension, and physical exercise per week	● Relatively easy to measure ● Contains few predictors	● No external validation
Yang and Zhang, 2021	Age, living alone, physical exercise, nutrition, and depression	● Relatively easy to measure ● Contains few predictors ● Performance is excellent ● Easy to implement in community settings	● No external validation ● Definition of CF varied in the development and validation datasets

IADL, instrumental activities of daily living scale; MoCA, montreal cognitive assessment; CDR, clinical dementia rating; TUG, timed-Up-and-Go; GDS, geriatric depression scale; NR, not reported; SNPs, single nucleotide polymorphisms.

## Discussion

This systematic review summarized and critically appraised eight prediction models on CF described in seven studies. Overall, most of them showed excellent or outstanding discrimination. However, there was still room for improvement. For example, the calibration and external validation could be conducted better.

It is worth noting that some models only included a few easy-to-obtain predictors with excellent performance (Yang and Zhang, 2021; Chen et al., 2022). Age, education, depression, and chronic diseases were the robust predictors consistent with previous findings (Yuan et al., 2022). Physical exercise was also a robust predictor. Studies have proven that physical exercise improves older adults’ cognitive function and physical status (Li et al., 2022). Liu et al. (2018) conducted a randomized controlled trial and confirmed that physical activity reduced the odds of worsening CF by 21%. Future studies are suggested to pay attention to an exercise intervention in older adults to prevent CF.

Modeling methods in these studies included logistic regression and machine learning. In general, machine learning models have advantages of flexibility, scalability, and better performance than logistic regression models (DeGregory et al., 2018; Ngiam and Khor, 2019), since logistic regression methods need specific data requirements or assumptions (Wu et al., 2021). However, in this study, the performance of machine learning models was worse than logistic regression models. The reason may be that the model’s predictive performance is influenced not only by the modeling approach but also by the methodological quality, which may lead to an inaccurate estimation of the prediction performance (Moons et al., 2019). However, the sample size needs to be much bigger while machine learning is performed (Moons et al., 2019). Therefore, the machine learning approaches are better suited for large sample sizes with numerous variables.

In this study, we found that most models were calibrated using the Hosmer–Lemeshow test. However, the *p*-value obtained by the test cannot be used to quantify calibration, so this test is not recommended (Steyerberg and Vergouwe, 2014). There is no best method to calibrate, and all methods have advantages and limitations (Huang et al., 2020). A calibration plot is the most commonly used method for visual displaying.

Models were rarely validated externally. Since only internal validation will lead to high prediction performance, external validation is required to quantify the prediction performance in other study populations for portability and generalization (Moons et al., 2012). Therefore, the external validation of the CF prediction model needs to be strengthened in the future.

All models were judged as high ROB, mainly due to the insufficient sample size, failure to handle missing data, selecting predictors by univariable analysis, and inappropriate ways to categorize continuous predictors, which may lead to an inaccurate estimation of prediction performances of the final model (Moons et al., 2019). Handling missing data by direct elimination will reduce the adequate sample size and increase the ROB of the prediction model. Multiple imputations are considered the best way to handle missing data because it allows users to explicitly incorporate the uncertainty about the actual value of imputed variables (Austin et al., 2021). Predictors selection based on univariable analysis is to independently analyze whether this factor has statistical significance on the occurrence of the outcome, usually measured by *p*-value. It may miss some essential variables and increase ROB because there may be some correlation between predictors and only after adjusting for other predictors to reach statistical significance (Moons et al., 2019). Although transforming continuous into categorical variables helps explain the results, it will lead to the loss of information and reduce the prediction performance (Collins et al., 2016). An EPV of more than 20 is less likely to have overfitting for model development, and a higher EPV is required (often > 200) when using the machine-learning technique (Moons et al., 2019).

We found some limitations in the geographical location of the models. Most models were developed among Asians. The study has suggested that CF in America and Europe are also prevalent, but rare models have been developed in these regions (Qiu et al., 2022). Since race disparities in the prevalence of cognitive impairment or frailty, tailored prediction models or external validation for American and European older adults are significant (Pandit et al., 2020; Wright et al., 2021).

### Strengths and limitations

In this study, we conducted a systematic literature search, extracted data in detail, applied PROBAST to evaluate the model, and provided advice for CF models’ future improvement. However, this study also has some limitations. Firstly, the results were not quantitatively synthesized because all models were judged as high ROB, and the assessment criteria of CF, types of predictors, modeling methods, and characteristics of participants varied. Secondly, we only included Chinese and English literature and did not retrieve the gray literature. These may result in an incomplete inclusion of CF prediction models. Lastly, geographical limitations existed because the included models were mainly developed based on Asians.

### Recommendations and implications

There are several suggestions for future research. Firstly, more prediction models should be developed based on a large sample size to make the results more accurate. Secondly, future studies should strictly follow the Transparent reporting of a multivariable prediction model for individual prognosis or diagnosis (TRIPOD) statement to standardize and transparent the studies of prediction models (Collins et al., 2015). Thirdly, more attention should be paid to improving the prediction performance and methodological quality, such as choosing appropriate predictors selection methods and modeling methods, using the method of multiple imputations to handle missing data, and validating internally and externally. Finally, it is necessary to evaluate the heterogeneity of prediction models for CF in different subpopulations by the individual participant data (IPD) meta-analysis so that prediction models can be customized for different subgroups (Damen et al., 2016).

## Conclusion

There are a few prediction models for CF. As a result of methodological shortcomings, incomplete presentation, and lack of external validation, the models’ usefulness still needs to be determined. In the future, models with better prediction performance and methodological quality should be developed and validated externally.

## Data availability statement

The original contributions presented in this study are included in the article/Supplementary material, further inquiries can be directed to the corresponding author.

## Author contributions

HF designed the study question. JH completed the literature search and wrote the draft. JH, XZ, and MH selected the articles, extracted the data, and assessed the quality. JH and XZ prepared the tables and figures. XZ, MH, HN, SW, RP, and HF revised the article, and provided critical recommendations on structure and presentation. All authors approved the final version of the manuscript.
