# Supplementary material for: Prediction model for cognitive frailty in older adults: A systematic review and critical appraisal
Source: Front Aging Neurosci. 2023 Apr 12;15:1119194. doi: 10.3389/fnagi.2023.1119194 (PMC10130444; doi:10.3389/fnagi.2023.1119194)
Supplement: Supplementary file 1 [file Table_1.docx]

**Prediction model for cognitive frailty in older adults: a systematic review and critical appraisal**

Supplementary materials:

PRISMA 2020 Checklist

Appendix 1: details of search strategies

Table 1: Detailed ROB assessment (using PROBAST) of cognitive frailty prediction models.

PRISMA 2020 Checklist

| **Section and Topic** | **Item #** | **Checklist item** | **Location where item is reported** |
| --- | --- | --- | --- |
| **TITLE** | | |  |
| Title | 1 | Identify the report as a systematic review. | Page 1 |
| **ABSTRACT** | | |  |
| Abstract | 2 | See the PRISMA 2020 for Abstracts checklist. | Page 2 |
| **INTRODUCTION** | | |  |
| Rationale | 3 | Describe the rationale for the review in the context of existing knowledge. | Page 2-3 |
| Objectives | 4 | Provide an explicit statement of the objective(s) or question(s) the review addresses. | Page 3 |
| **METHODS** | | |  |
| Eligibility criteria | 5 | Specify the inclusion and exclusion criteria for the review and how studies were grouped for the syntheses. | Page 3 |
| Information sources | 6 | Specify all databases, registers, websites, organisations, reference lists and other sources searched or consulted to identify studies. Specify the date when each source was last searched or consulted. | Page 3-4 |
| Search strategy | 7 | Present the full search strategies for all databases, registers and websites, including any filters and limits used. | Appendix 1 |
| Selection process | 8 | Specify the methods used to decide whether a study met the inclusion criteria of the review, including how many reviewers screened each record and each report retrieved, whether they worked independently, and if applicable, details of automation tools used in the process. | Page 4 |
| Data collection process | 9 | Specify the methods used to collect data from reports, including how many reviewers collected data from each report, whether they worked independently, any processes for obtaining or confirming data from study investigators, and if applicable, details of automation tools used in the process. | Page 4-5 |
| Data items | 10a | List and define all outcomes for which data were sought. Specify whether all results that were compatible with each outcome domain in each study were sought (e.g. for all measures, time points, analyses), and if not, the methods used to decide which results to collect. | Page 4-5 |
|  | 10b | List and define all other variables for which data were sought (e.g. participant and intervention characteristics, funding sources). Describe any assumptions made about any missing or unclear information. | Page 4-5 |
| Study risk of bias assessment | 11 | Specify the methods used to assess risk of bias in the included studies, including details of the tool(s) used, how many reviewers assessed each study and whether they worked independently, and if applicable, details of automation tools used in the process. | Page 5 |
| Effect measures | 12 | Specify for each outcome the effect measure(s) (e.g. risk ratio, mean difference) used in the synthesis or presentation of results. | Page 8 |
| Synthesis methods | 13a | Describe the processes used to decide which studies were eligible for each synthesis (e.g. tabulating the study intervention characteristics and comparing against the planned groups for each synthesis (item #5)). | Page 55 |
|  | 13b | Describe any methods required to prepare the data for presentation or synthesis, such as handling of missing summary statistics, or data conversions. | Not applicable |
|  | 13c | Describe any methods used to tabulate or visually display results of individual studies and syntheses. | Not applicable |
|  | 13d | Describe any methods used to synthesize results and provide a rationale for the choice(s). If meta-analysis was performed, describe the model(s), method(s) to identify the presence and extent of statistical heterogeneity, and software package(s) used. | Not applicable |
|  | 13e | Describe any methods used to explore possible causes of heterogeneity among study results (e.g. subgroup analysis, meta-regression). | Not applicable |
|  | 13f | Describe any sensitivity analyses conducted to assess robustness of the synthesized results. | Not applicable |
| Reporting bias assessment | 14 | Describe any methods used to assess risk of bias due to missing results in a synthesis (arising from reporting biases). | Not applicable |
| Certainty assessment | 15 | Describe any methods used to assess certainty (or confidence) in the body of evidence for an outcome. | Not applicable |
| **RESULTS** | | |  |
| Study selection | 16a | Describe the results of the search and selection process, from the number of records identified in the search to the number of studies included in the review, ideally using a flow diagram. | Page 5 |
|  | 16b | Cite studies that might appear to meet the inclusion criteria, but which were excluded, and explain why they were excluded. | Figure 1 |
| Study characteristics | 17 | Cite each included study and present its characteristics. | Page 5 and Tables 1 and 2 |
| Risk of bias in studies | 18 | Present assessments of risk of bias for each included study. | Page 8, Table 3 and supplementary Table 1 |
| Results of individual studies | 19 | For all outcomes, present, for each study: (a) summary statistics for each group (where appropriate) and (b) an effect estimate and its precision (e.g. confidence/credible interval), ideally using structured tables or plots. | Tables 1 and 2 |
| Results of syntheses | 20a | For each synthesis, briefly summarise the characteristics and risk of bias among contributing studies. | Not applicable |
|  | 20b | Present results of all statistical syntheses conducted. If meta-analysis was done, present for each the summary estimate and its precision (e.g. confidence/credible interval) and measures of statistical heterogeneity. If comparing groups, describe the direction of the effect. | Not applicable |
|  | 20c | Present results of all investigations of possible causes of heterogeneity among study results. | Not applicable |
|  | 20d | Present results of all sensitivity analyses conducted to assess the robustness of the synthesized results. | Not applicable |
| Reporting biases | 21 | Present assessments of risk of bias due to missing results (arising from reporting biases) for each synthesis assessed. | Tables 1 and 3 |
| Certainty of evidence | 22 | Present assessments of certainty (or confidence) in the body of evidence for each outcome assessed. | Not applicable |
| **DISCUSSION** | | |  |
| Discussion | 23a | Provide a general interpretation of the results in the context of other evidence. | Page 8 |
|  | 23b | Discuss any limitations of the evidence included in the review. | Page 8-9 |
|  | 23c | Discuss any limitations of the review processes used. | Page 10 |
|  | 23d | Discuss implications of the results for practice, policy, and future research. | Page 10 |
| **OTHER INFORMATION** | | |  |
| Registration and protocol | 24a | Provide registration information for the review, including register name and registration number, or state that the review was not registered. | Page 3 |
|  | 24b | Indicate where the review protocol can be accessed, or state that a protocol was not prepared. | Page 3 |
|  | 24c | Describe and explain any amendments to information provided at registration or in the protocol. | Page 3 |
| Support | 25 | Describe sources of financial or non-financial support for the review, and the role of the funders or sponsors in the review. | Page 10 |
| Competing interests | 26 | Declare any competing interests of review authors. | Page 10 |
| Availability of data, code and other materials | 27 | Report which of the following are publicly available and where they can be found: template data collection forms; data extracted from included studies; data used for all analyses; analytic code; any other materials used in the review. | Not report |

*From:*  Page MJ, McKenzie JE, Bossuyt PM, Boutron I, Hoffmann TC, Mulrow CD, et al. The PRISMA 2020 statement: an updated guideline for reporting systematic reviews. BMJ 2021;372:n71. doi: 10.1136/bmj.n71

Appendix 1: details of search strategies.

Pubmed

| Number | Search strategy | results |
| --- | --- | --- |
| #1 | elder*[Title/Abstract] | 288,201 |
| #2 | old*[Title/Abstract] | 1,164,345 |
| #3 | #1 OR #2 | 1,415,064 |
| #4 | cognitive frailty[Title/Abstract] | 239 |
| #5 | "Cognitive Dysfunction"[Mesh] | 28,678 |
| #6 | cognitive impairment[Title/Abstract] | 70,918 |
| #7 | cognitive decline[Title/Abstract] | 27,710 |
| #8 | #4 OR #5 OR #6 OR #7 | 97,946 |
| #9 | "Frailty"[Mesh] | 6,026 |
| #10 | frail*[Title/Abstract] | 29,569 |
| #11 | frailty syndrome[Title/Abstract] | 608 |
| #12 | pre-frail*[Title/Abstract] | 1,140 |
| #13 | #9 OR #10 OR #11 OR #12 | 29,905 |
| #14 | "Clinical Decision Rules"[Mesh] | 846 |
| #15 | clinical decision rules[Title/Abstract] | 570 |
| #16 | prediction model[Title/Abstract] | 17,374 |
| #17 | clinical prediction[Title/Abstract] | 3,507 |
| #18 | predictive model[Title/Abstract] | 12,880 |
| #19 | predictive scor*[Title/Abstract] | 1,514 |
| #20 | prediction scor*[Title/Abstract] | 2,038 |
| #21 | predictive risk[Title/Abstract] | 1,740 |
| #22 | prediction risk[Title/Abstract] | 121 |
| #23 | risk scor*[Title/Abstract] | 29,787 |
| #24 | predictive value[Title/Abstract] | 104,902 |
| #25 | prediction value[Title/Abstract] | 661 |
| #26 | #14 OR #15 OR #16 OR #17 OR #18 OR #19 OR #20 OR #21 OR #22 OR #23 OR #24 OR #25 | 166,360 |
| #27 | #3 AND #8 AND #13 AND #26 | 18 |

Embase

| Number | Search strategy | | results |
| --- | --- | --- | --- |
| #1 | | elder*:ab,ti | 400257 |
| #2 | | old*:ab,ti | 2332900 |
| #3 | | #1 OR #2 | 2603488 |
| #4 | | 'cognitive frailty':ab,ti | 273 |
| #5 | | 'cognitive dysfunction'/exp | 555804 |
| #6 | | 'cognitive impairment':ab,ti | 106437 |
| #7 | | 'cognitive decline':ab,ti | 41529 |
| #8 | | #4 OR #5 OR #6 OR #7 | 576196 |
| #9 | | 'frailty'/exp | 18540 |
| #10 | | Frail*:ab,ti | 43035 |
| #11 | | 'pre frail*':ab,ti | 1867 |
| #12 | | 'frailty syndrome':ab,ti | 887 |
| #13 | | #9 OR #10 OR #11 OR #12 | 45492 |
| #14 | | 'clinical decision rule'/exp | 430 |
| #15 | | 'clinical decision rule':ab,ti | 765 |
| #16 | | 'prediction model':ab,ti | 22862 |
| #17 | | 'clinical prediction':ab,ti | 4674 |
| #18 | | 'predictive scor*':ab,ti | 2447 |
| #19 | | 'prediction scor*':ab,ti | 3420 |
| #20 | | 'predictive model':ab,ti | 17904 |
| #21 | | 'prediction risk':ab,ti | 176 |
| #22 | | 'predictive risk':ab,ti | 2722 |
| #23 | | 'risk scor*':ab,ti | 51013 |
| #24 | | 'predictive value':ab,ti | 158134 |
| #25 | | 'prediction value':ab,ti | 1090 |
| #26 | | #14 OR #15 OR #16 OR #17 OR #18 OR #19 OR #20 OR #21 OR #22 OR #23 OR #24 OR #25 | 251108 |
| #27 | | #3 AND #8 AND #13 AND #26 | 104 |

Web of science

| Number | Search strategy | | results |
| --- | --- | --- | --- |
| #1 | | TS=(elder*) | 508535 |
| #2 | | TS=(old*) | 2518042 |
| #3 | | #2 OR #1 | 2818339 |
| #4 | | TS=(cognitive frailty) | 4450 |
| #5 | | TS=(cognitive dysfunction) | 90842 |
| #6 | | TS=(cognitive impairment) | 195092 |
| #7 | | TS=(cognitive decline) | 68011 |
| #8 | | #4 OR #5 OR #6 OR #7 | 266092 |
| #9 | | TS=(frailty) | 32843 |
| #10 | | TS=(frail*) | 50409 |
| #11 | | TS=(pre-frail*) | 1380 |
| #12 | | TS=(frailty syndrome) | 4914 |
| #13 | | #9 OR #10 OR #11 OR #12 | 50409 |
| #14 | | TS=(clinical decision rules) | 11473 |
| #15 | | TS=(prediction model) | 879490 |
| #16 | | TS=(clinical prediction) | 216484 |
| #17 | | TS=(predictive model) | 327657 |
| #18 | | TS=(predictive scor*) | 109632 |
| #19 | | TS=(prediction scor*) | 92959 |
| #20 | | TS=(predictive risk) | 230102 |
| #21 | | TS=(prediction risk) | 180536 |
| #22 | | TS=(risk scor*) | 423379 |
| #23 | | TS=(predictive value) | 408177 |
| #24 | | TS=(prediction value) | 293494 |
| #25 | | #24 OR #23 OR #22 OR #21 OR #20 OR #19 OR #18 OR #17 OR #16 OR #15 OR #14 | 2001545 |
| #26 | | #13 AND #3 AND #8 AND #25 | 1144 |

The Cochrane library

| Number | Search strategy | | results |
| --- | --- | --- | --- |
| #1 | | (elder*):ti,ab,kw | 55499 |
| #2 | | (old*):ti,ab,kw | 121911 |
| #3 | | #1 or #2 | 163113 |
| #4 | | MeSH descriptor: [Cognitive Dysfunction] explode all trees | 2115 |
| #5 | | (cognitive frailty):ti,ab,kw | 517 |
| #6 | | (cognitive impairment):ti,ab,kw | 13192 |
| #7 | | (cognitive decline):ti,ab,kw | 5319 |
| #8 | | #4 or #5 or #6 or #7 | 17665 |
| #9 | | MeSH descriptor: [Frailty] explode all trees | 249 |
| #10 | | (frail*):ti,ab,kw | 4359 |
| #11 | | (frailty syndrome):ti,ab,kw | 250 |
| #12 | | (pre-frail*):ti,ab,kw | 303 |
| #13 | | #9 or #10 or #11 or #12 | 4359 |
| #14 | | MeSH descriptor: [Clinical Decision Rules] explode all trees | 19 |
| #15 | | (clinical decision rules):ti,ab,kw | 335 |
| #16 | | (prediction model):ti,ab,kw | 4848 |
| #17 | | (clinical prediction):ti,ab,kw | 10770 |
| #18 | | (predictive model):ti,ab,kw | 6060 |
| #19 | | (predictive score*):ti,ab,kw | 6853 |
| #20 | | (prediction scor*):ti,ab,kw | 3818 |
| #21 | | (predictive risk):ti,ab,kw | 10033 |
| #22 | | (prediction risk):ti,ab,kw | 5458 |
| #23 | | (risk scor*):ti,ab,kw | 45387 |
| #24 | | (predictive value):ti,ab,kw | 16151 |
| #25 | | (prediction value):ti,ab,kw | 3583 |
| #26 | | #14 OR #15 OR #16 OR #17 OR #18 OR #19 OR #20 OR #21 OR #22 OR #23 OR #24 OR #25 | 75225 |
| #27 | | #3 AND #8 AND #13 AND #26 in trails | 93 |

Scopus

| Number | Search strategy | Results |
| --- | --- | --- |
| #1 | TITLE-AB-KEY: elder* OR old* | 3284890 |
| #2 | TITLE-AB-KEY: "cognitive frailty" OR "cognitive dysfunction" OR "cognitive impairment" OR "cognitive decline" | 153291 |
| #3 | TITLE-AB-KEY: frailty OR Frail* OR "pre frail*" OR "frailty syndrome" | 50612 |
| #4 | TITLE-AB-KEY: "clinical decision rule" OR "clinical decision rule" OR "prediction model" OR "clinical prediction" OR "predictive scor*" OR "prediction scor*" OR "predictive model" OR "prediction risk" OR "predictive risk" OR "risk scor*" OR "predictive value" OR "prediction value" | 625518 |
| #5 | #1 AND #2 AND #3 AND #4 | 118 |

APA PsycINFO

| Number | Search strategy | Results |
| --- | --- | --- |
| #1 | TX: elder* OR old* | 338718 |
| #2 | TX: "cognitive frailty" OR "cognitive dysfunction" OR "cognitive impairment" OR "cognitive decline" | 31510 |
| #3 | TX: frailty OR Frail* OR "pre frail*" OR "frailty syndrome" | 12771 |
| #4 | TX: "clinical decision rule" OR "clinical decision rule" OR "prediction model" OR "clinical prediction" OR "predictive scor*" OR "prediction scor*" OR "predictive model" OR "prediction risk" OR "predictive risk" OR "risk scor*" OR "predictive value" OR "prediction value" | 39498 |
| #5 | #1 AND #2 AND #3 AND #4 | 19 |

CINAHL

| Number | Search strategy | Results |
| --- | --- | --- |
| #1 | AB: elder* OR old* | 2407874 |
| #2 | AB: "cognitive frailty" OR "cognitive dysfunction" OR "cognitive impairment" OR "cognitive decline" | 773483 |
| #3 | AB: frailty OR Frail* OR "pre frail*" OR "frailty syndrome" | 66896 |
| #4 | AB: "clinical decision rule" OR "clinical decision rule" OR "prediction model" OR "clinical prediction" OR "predictive scor*" OR "prediction scor*" OR "predictive model" OR "prediction risk" OR "predictive risk" OR "risk scor*" OR "predictive value" OR "prediction value" | 229091 |
| #5 | #1 AND #2 AND #3 AND #4 | 11 |

Wanfang databases

| Number | Search strategy | | Results |
| --- | --- | --- | --- |
| #1 | | 主题：老年人 | 426918 |
| #2 | | 主题：认知衰弱 or 认知衰退 | 75 |
| #3 | | 主题：风险预测模型 or 风险预测评分 or 风险预测工具 or 预测指数 | 90487 |
| #4 | | #1 AND #2 AND #3 | 23 |

CNKI

| Number | Search strategy | | results |
| --- | --- | --- | --- |
| #1 | | 主题：老年人 | 285621 |
| #2 | | 主题：认知衰弱 + 认知衰退 | 370 |
| #3 | | 主题：风险预测模型 + 风险预测评分 + 风险预测工具 + 预测指数 | 6679 |
| #4 | | #1 * #2 * #3 | 4 |

Table 3 Detailed ROB assessment (using PROBAST) of cognitive frailty prediction models.

| Studies | 1.1 | 1.2 | 2.1 | 2.2 | 2.3 | 3.1 | 3.2 | 3.3 | 3.4 | 3.5 | 3.6 | 4.1 | 4.2 | 4.3 | 4.4 | 4.5 | 4.6 | 4.7 | 4.8 | 4.9 |
| --- | --- | --- | --- | --- | --- | --- | --- | --- | --- | --- | --- | --- | --- | --- | --- | --- | --- | --- | --- | --- |
| Chen2022 | Y | Y | Y | Y | Y | Y | Y | Y | Y | Y | Y | N | N | Y | Y | N | Y | Y | Y | PY |
| Navarro-Pardo 2020 | Y | Y | Y | Y | Y | Y | Y | Y | Y | Y | Y | N | N | Y | Y | N | NI | N | NI | PY |
| Rivan2020 | Y | Y | Y | Y | Y | Y | Y | PN | Y | Y | Y | N | Y | Y | N | N | PN | PY | NI | PY |
| Sargent2020(1) | Y | Y | Y | Y | Y | Y | Y | Y | Y | Y | Y | N | Y | N | N | PY | Y | Y | Y | PY |
| Sargent2020(2) | Y | Y | Y | Y | Y | Y | Y | Y | Y | Y | Y | N | Y | N | N | PY | Y | Y | Y | PY |
| Tseng2019 | Y | Y | Y | Y | Y | Y | Y | Y | Y | Y | Y | PY | Y | PY | Y | N | NI | Y | Y | Y |
| Wen2021 | Y | Y | Y | Y | Y | Y | Y | Y | Y | Y | Y | PY | N | N | N | N | Y | Y | Y | PY |
| Yang2021 | Y | Y | Y | Y | Y | Y | Y | Y | Y | Y | Y | Y | N | N | N | N | Y | Y | Y | PY |

ROB = risk of bias; PROBAST = Prediction model risk of bias assessment tool. Y = YES; PY = PROBABLY YES; NI = NO INFORMATION; PN = PROBABLY NO; N = NO

Notes: 1.1 Were appropriate data sources used, for example, cohort, RCT, or nested case-control study data? 1.2 Were all inclusions and exclusions of participants appropriate? 2.1 Were predictors defined and assessed in a similar way for all participants? 2.2 Were predictor assessments made without knowledge of outcome data? 2.3 Are all predictors available at the time the model is intended to be used? 3.1 Was the outcome determined appropriately? 3.2 Was a prespecified or standard outcome definition used? 3.3 Were predictors excluded from the outcome definition? 3.4 Was the outcome defined and determined in a similar way for all participants? 3.5 Was the outcome determined without knowledge of predictor information? 3.6 Was the time interval between predictor assessment and outcome determination appropriate? 4.1 Were there a reasonable number of participants with the outcome? 4.2 Were continuous and categorical predictors handled appropriately? 4.3 Were all enrolled participants included in the analysis? 4.4 Were participants with missing data handled appropriately? 4.5 Was selection of predictors based on univariable analysis avoided? (For development model) 4.6 Were complexities in the data accounted for appropriately? 4.7 Were relevant model performance measures evaluated appropriately? 4.8 Were model overfitting, underfitting, and optimism in model performance accounted for? (For development model) 4.9 Do predictors and their assigned weights in the final model correspond to the results from the reported multivariable analysis? (For development model).
